# Supplementary material for: Transcriptional rewiring over evolutionary timescales changes quantitative and qualitative properties of gene expression
Source: eLife. 2016 Sep 10;5:e18981. doi: 10.7554/eLife.18981 (PMC5067116; doi:10.7554/eLife.18981)
Supplement: Figure 3—source data 1. — Colonies observed on galactose + Antimycin A and glucose + Antimycin A are tabulated for 212 strains, each one being a knockout of an individual transcriptional regulator. This data is plotted in Figure 3B. DOI: http://dx.doi.org/10.7554/eLife.18981.007 [file elife-18981-fig3-data1.docx]

**Figure3- Source Data: TFKO Antimycin A spotting results**

| **TFKO number** | **ORF** | **Name** | **Colonies Observed- Galactose+ Antimycin A** | **Colonies Observed- Glucose + Antimycin A** |
| --- | --- | --- | --- | --- |
| TF001 | orf19.454 | SFL1 | 5 | 5 |
| TF002 | orf19.921 | orf19.921 | 5 | 5 |
| TF003 | orf19.1069 | RPN4 | 5 | 5 |
| TF004 | orf19.1253 | orf19.1253 | 5 | 5 |
| TF005 | orf19.1499 | orf19.1499 | 5 | 5 |
| TF006 | orf19.2646 | orf19.2646 | 5 | 5 |
| TF007 | orf19.2647 | orf19.2647 | 5 | 5 |
| TF008 | orf19.2730 | orf19.2730 | 5 | 4 |
| TF009 | orf19.2748 | orf19.2748 | 5 | 5 |
| TF010 | orf19.3188 | TAC1 | 5 | 5 |
| TF011 | orf19.3190 | HAL9 | 5 | 5 |
| TF012 | orf19.3305 | orf19.3305 | 5 | 5 |
| TF013 | orf19.3308 | orf19.3308 | 5 | 5 |
| TF014 | orf19.3434 | orf19.3434 | 5 | 5 |
| TF015 | orf19.3753 | SEF1 | 4 | 3 |
| TF016 | orf19.3809 | orf19.3809 | 5 | 5 |
| TF017 | orf19.3876 | orf19.3876 | 5 | 5 |
| TF018 | orf19.3912 | GLN3 | 5 | 5 |
| TF019 | orf19.3928 | orf19.3928 | 5 | 5 |
| TF020 | orf19.3969 | orf19.3969 | 5 | 5 |
| TF021 | orf19.4000 | orf19.4000 | 5 | 5 |
| TF022 | orf19.4056 | BRG1 | 5 | 5 |
| TF023 | orf19.4145 | orf19.4145 | 5 | 5 |
| TF024 | orf19.4166 | orf19.4166 | 5 | 5 |
| TF025 | orf19.4225 | orf19.4225 | 5 | 5 |
| TF026 | orf19.4251 | orf19.4251 | 5 | 5 |
| TF027 | orf19.4288 | CTA7 | 4 | 4 |
| TF028 | orf19.4438 | RME1 | 5 | 5 |
| TF029 | orf19.4450 | orf19.4450 | 5 | 5 |
| TF030 | orf19.4524 | orf19.4524 | 5 | 5 |
| TF031 | orf19.4568 | ZCF25 | 5 | 5 |
| TF032 | orf19.4662 | RLM1 | 5 | 5 |
| TF033 | orf19.4670 | CAS5 | 4 | 4 |
| TF034 | orf19.4722 | orf19.4722 | 0 | 5 |
| TF035 | orf19.4778 | orf19.4778 | 5 | 5 |
| TF036 | orf19.4853 | HCM1 | 5 | 5 |
| TF037 | orf19.4941 | TYE7 | 5 | 5 |
| TF038 | orf19.4972 | orf19.4972 | 5 | 5 |
| TF039 | orf19.5001 | CUP2 | 5 | 5 |
| TF040 | orf19.5026 | orf19.5026 | 5 | 5 |
| TF041 | orf19.5097 | CAT8 | 5 | 5 |
| TF042 | orf19.5133 | orf19.5133 | 5 | 5 |
| TF043 | orf19.5251 | orf19.5251 | 5 | 5 |
| TF044 | orf19.5326 | orf19.5326 | 5 | 5 |
| TF045 | orf19.5338 | GAL4 | 5 | 5 |
| TF046 | orf19.5380 | orf19.5380 | 5 | 5 |
| TF047 | orf19.5498 | EFH1 | 5 | 5 |
| TF048 | orf19.5548 | orf19.5548 | 5 | 5 |
| TF049 | orf19.5651 | orf19.5651 | 5 | 5 |
| TF050 | orf19.5849 | CWT1 | 5 | 5 |
| TF051 | orf19.5855 | orf19.5855 | 5 | 4 |
| TF052 | orf19.5917 | STP3 | 5 | 5 |
| TF053 | orf19.5924 | orf19.5924 | 5 | 5 |
| TF054 | orf19.5940 | orf19.5940 | 5 | 5 |
| TF055 | orf19.5975 | orf19.5975 | 5 | 5 |
| TF056 | orf19.5992 | WOR2 | 5 | 5 |
| TF057 | orf19.6038 | orf19.6038 | 5 | 5 |
| TF058 | orf19.6102 | orf19.6102 | 5 | 4 |
| TF059 | orf19.6124 | ACE2 | 5 | 5 |
| TF060 | orf19.6182 | orf19.6182 | 5 | 5 |
| TF061 | orf19.6514 | CUP9 | 5 | 5 |
| TF062 | orf19.6824 | orf19.6824 | 5 | 5 |
| TF063 | orf19.6874 | orf19.6874 | 5 | 4 |
| TF064 | orf19.7017 | orf19.7017 | 5 | 5 |
| TF065 | orf19.7068 | MAC1 | 5 | 5 |
| TF066 | orf19.7317 | orf19.7317 | 5 | 5 |
| TF067 | orf19.7319 | SUC1 | 5 | 5 |
| TF068 | orf19.7359 | CRZ1 | 5 | 5 |
| TF069 | orf19.7372 | MRR1 | 5 | 5 |
| TF070 | orf19.7374 | CTA4 | 5 | 4 |
| TF071 | orf19.7518 | orf19.7518 | 5 | 5 |
| TF072 | orf19.7570 | orf19.7570 | 5 | 5 |
| TF073 | orf19.7583 | orf19.7583 | 5 | 4 |
| TF074 | orf19.166 | orf19.166 | 5 | 5 |
| TF075 | orf19.217 | orf19.217 | 4 | 5 |
| TF076 | orf19.255 | orf19.255 | 5 | 5 |
| TF077 | orf19.391 | UPC2 | 5 | 5 |
| TF078 | orf19.431 | orf19.431 | 5 | 5 |
| TF079 | orf19.517 | HAP31 | 5 | 5 |
| TF080 | orf19.681 | HAP43 | 5 | 5 |
| TF081 | orf19.837.1 | INO4 | 5 | 5 |
| TF082 | orf19.909 | STP4 | 5 | 5 |
| TF083 | orf19.971 | SKN7 | 5 | 5 |
| TF084 | orf19.1032 | SKO1 | 5 | 5 |
| TF085 | orf19.1035 | WAR1 | 5 | 5 |
| TF086 | orf19.1168 | orf19.1168 | 5 | 5 |
| TF087 | orf19.1228 | orf19.1228 | 5 | 5 |
| TF088 | orf19.1274 | orf19.1274 | 5 | 5 |
| TF089 | orf19.1496 | orf19.1496 | 5 | 5 |
| TF090 | orf19.1543 | OPI1 | 5 | 5 |
| TF091 | orf19.1685 | orf19.1685 | 5 | 5 |
| TF092 | orf19.1926 | SEF2 | 5 | 5 |
| TF093 | orf19.1973 | HAP5 | 5 | 5 |
| TF094 | orf19.2088 | orf19.2088 | 5 | 5 |
| TF095 | orf19.2119 | NDT80B | 5 | 5 |
| TF096 | orf19.2356 | CRZ2 | 5 | 5 |
| TF097 | orf19.2476 | orf19.2476 | 5 | 5 |
| TF098 | orf19.2745 | orf19.2745 | 5 | 5 |
| TF099 | orf19.2753 | orf19.2753 | 5 | 5 |
| TF100 | orf19.2808 | orf19.2808 | 5 | 5 |
| TF101 | orf19.2842 | GZF3 | 5 | 5 |
| TF102 | orf19.2961 | orf19.2961 | 5 | 5 |
| TF103 | orf19.3063 | orf19.3063 | 5 | 5 |
| TF104 | orf19.3127 | CZF1 | 5 | 5 |
| TF105 | orf19.3625 | orf19.3625 | 5 | 5 |
| TF106 | orf19.3794 | ZAP1 | 5 | 4 |
| TF107 | orf19.4318 | MIG1 | 5 | 5 |
| TF108 | orf19.4647 | HAP3 | 5 | 5 |
| TF109 | orf19.4752 | MSN4 | 5 | 5 |
| TF110 | orf19.4998 | ROB1 | 5 | 5 |
| TF111 | orf19.5249 | orf19.5249 | 5 | 5 |
| TF112 | orf19.5343 | ASH1 | 5 | 5 |
| TF113 | orf19.5558 | RBF1 | 5 | 5 |
| TF114 | orf19.5729 | FGR17 | 5 | 5 |
| TF115 | orf19.5908 | TEC1 | 5 | 5 |
| TF116 | orf19.5910 | orf19.5910 | 5 | 5 |
| TF117 | orf19.6109 | TUP1 | 5 | 5 |
| TF118 | orf19.6121 | orf19.6121 | 5 | 5 |
| TF119 | orf19.6680 | FGR27 | 5 | 5 |
| TF120 | orf19.6781 | orf19.6781 | 5 | 5 |
| TF121 | orf19.6798 | SSN6 | 5 | 5 |
| TF122 | orf19.6817 | FCR1 | 5 | 5 |
| TF123 | orf19.6888 | orf19.6888 | 4 | 5 |
| TF124 | orf19.6985 | TEA1 | 5 | 5 |
| TF125 | orf19.7150 | NRG1 | 5 | 5 |
| TF126 | orf19.7247 | RIM101 | 5 | 5 |
| TF127 | orf19.7401 | ISW2 | 5 | 5 |
| TF128 | orf19.7436 | AAF1 | 5 | 5 |
| TF129 | orf19.3193 | FCR3 | 5 | 5 |
| TF130 | orf19.4766 | orf19.4766 | 5 | 5 |
| TF131 | orf19.7371 | orf19.7371 | 5 | 5 |
| TF132 | orf19.7381 | AHR1 | 5 | 5 |
| TF133 | orf19.3187 | ZNC1 | 5 | 5 |
| TF134 | orf19.4545 | SWI4 | 4 | 5 |
| TF135 | orf19.4573 | orf19.4573 | 5 | 5 |
| TF136 | orf19.173 | orf19.173 | 5 | 5 |
| TF137 | orf19.723 | BCR1 | 5 | 5 |
| TF138 | orf19.1187 | CPH2 | 5 | 5 |
| TF139 | orf19.1275 | GAT1 | 5 | 5 |
| TF140 | orf19.1623 | CAP1 | 4 | 4 |
| TF141 | orf19.1718 | orf19.1718 | 5 | 5 |
| TF142 | orf19.2315 | orf19.2315 | 0 | 5 |
| TF143 | orf19.2612 | orf19.2612 | 5 | 5 |
| TF144 | orf19.3182 | GIS2 | 5 | 5 |
| TF145 | orf19.3736 | KAR4 | 5 | 5 |
| TF146 | orf19.4767 | orf19.4767 | 5 | 5 |
| TF147 | orf19.4869 | SFU1 | 5 | 5 |
| TF148 | orf19.1227 | orf19.1227 | 5 | 5 |
| TF149 | orf19.1255 | orf19.1255 | 5 | 5 |
| TF150 | orf19.1497 | orf19.1497 | 5 | 5 |
| TF151 | orf19.1577 | orf19.1577 | 4 | 4 |
| TF152 | orf19.2054 | FGR15 | 5 | 5 |
| TF153 | orf19.3012 | ARO80 | 5 | 5 |
| TF154 | orf19.4649 | orf19.4649 | 5 | 5 |
| TF155 | orf19.3252 | DAL81 | 5 | 5 |
| TF156 | orf19.610 | EFG1 | 5 | 5 |
| TF157 | orf19.1150 | orf19.1150 | 5 | 5 |
| TF158 | orf19.1773 | RAP1 | 5 | 5 |
| TF159 | orf19.1757 | orf19.1757 | 4 | 5 |
| TF160 | orf19.2743 | orf19.2743 | 5 | 4 |
| TF161 | orf19.4776 | orf19.4776 | 5 | 5 |
| TF162 | orf19.4961 | STP2 | 4 | 4 |
| TF163 | orf19.3865 | orf19.3865 | 4 | 4 |
| TF164 | orf19.2747 | RGT1 | 4 | 4 |
| TF165 | orf19.3986 | orf19.3986 | 5 | 5 |
| TF166 | orf19.2823 | RFG1 | 5 | 4 |
| TF167 | orf19.7521 | orf19.7521 | 4 | 5 |
| TF168 | orf19.5210 | orf19.5210 | 4 | 4 |
| TF169 | orf19.4342 | orf19.4342 | 4 | 4 |
| TF170 | orf19.467 | WOR3 | 5 | 5 |
| TF171 | orf19.2623 | orf19.2623 | 4 | 5 |
| TF172 | orf19.1604 | orf19.1604 | 4 | 4 |
| TF173 | orf19.4590 | RFX2 | 5 | 5 |
| TF174 | orf19.4231 | PTH2 | 5 | 5 |
| TF175 | orf19.1093 | FLO8 | 4 | 5 |
| TF176 | orf19.4884 | WOR1 | 5 | 5 |
| TF177 | orf19.3048 | orf19.3048 | 4 | 4 |
| TF178 | orf19.513 | orf19.513 | 4 | 4 |
| TF179 | orf19.1822 | UME6 | 5 | 5 |
| TF180 | orf19.2272 | orf19.2272 | 4 | 5 |
| TF181 | orf19.6713 | orf19.6713 | 4 | 5 |
| TF182- this is a heterozygote | orf19.2876 | CBF1 | 4 | 4 |
| TF183 | orf19.4433 | CPH1 | 5 | 5 |
| TF184 | orf19.1358 | GCN4 | 4 | 4 |
| TF185 | orf19.7539 | INO2 | 4 | 5 |
| TF186 | orf19.2064 | orf19.2064 | 5 | 5 |
| TF187 | orf19.3845 | FGR3 | 5 | 5 |
| TF188 | orf19.2752 | ADR1 | 5 | 5 |
| TF189 | orf19.7397 | orf19.7397 | 5 | 5 |
| TF190 | orf19.7098 | orf19.7098 | 5 | 5 |
| TF191 | orf19.3722 | orf19.3722 | 5 | 5 |
| TF192 | orf19.4195 | orf19.4195 | 5 | 4 |
| TF193 | orf19.6173 | STD1 | 4 | 4 |
| TF194 | orf19.1481 | HAP42 | 5 | 4 |
| TF195 | orf19.1715 | IRO1 | 5 | 5 |
| TF196 | orf19.740 | HAP41 | 4 | 5 |
| TF197 | orf19.2432 | HAC1 | 4 | 5 |
| TF198 | orf19.6626 | orf19.6626 | 4 | 5 |
| TF199 | orf19.2077 | ZCF9 | 4 | 5 |
| TF200 | orf19.3088 | orf19.3088 | 4 | 4 |
| TF201 | orf19.2674 | orf19.2674 | 4 | 4 |
| TF202 | orf19.2736 | HFL2 | 4 | 4 |
| TF203 | orf19.7301 | orf19.7301 | 4 | 5 |
| TF205 | orf19.2458 | SIP5 | 4 | 4 |
| TF207 | orf19.6845 | orf19.6845 | 4 | 4 |
| TF208 | orf19.6168 | orf19.6168 | 4 | 4 |
| TF209 | orf19.4911 | orf19.4911 | 4 | 5 |
| TF210 | orf19.2131 | orf19.2131 | 4 | 4 |
| TF211 | orf19.2280 | ZCF10 | 5 | 5 |
| TF212 | orf19.1897 | orf19.1897 | 4 | 4 |
| TF213 | orf19.3405 | orf19.3405 | 4 | 4 |
| TF214 | orf19.1729 | orf19.1729 | 5 | 4 |
