# Supplementary material for: Transcriptional rewiring over evolutionary timescales changes quantitative and qualitative properties of gene expression
Source: eLife. 2016 Sep 10;5:e18981. doi: 10.7554/eLife.18981 (PMC5067116; doi:10.7554/eLife.18981)
Supplement: Supplementary file 1. — The clades, anatomical sites of infection, geographical sites of isolation and types of infection are listed for each isolate. DOI: http://dx.doi.org/10.7554/eLife.18981.023 [file elife-18981-supp1.docx]

**Supplementary File 1: *C. albicans* isolates used in Figure 4 figure supplement 2**

| **Strain name** | **Column in Figure 4 figure supp 2** | **Anatomical Origin** | **Type of infection** | **Geographical Location (Country)** | **Clade*** | **Reference** |
| --- | --- | --- | --- | --- | --- | --- |
| CEC2021 | 1 | blood | invasive | France | 2 | [1] |
| P37037 | 2 | oral | commensal | USA | I | [2] [3] |
| P75016 | 3 | blood | invasive | Israel | SA | [2] [3] |
| P57055 | 4 | blood | invasive | USA | III | [2] [3] |
| SC5314 | 5 |  | invasive |  | 1 | [4] [5] |
| CEC2020 | 6 | vagina | commensal | Morocco | 1 | [6] |
| CEC2022 | 7 | lung | invasive | France | 4 | [6] |
| CEC2023 | 8 | stool | commensal | French Guiana | 8 | [7] |
| CEC2018 | 9 | urine | superficial | France | 9 | [1] |
| CEC3494 | 10 | oral | commensal | Belgium | 11 | [1] |
| CEC1424 | 11 | oral | superficial | Nigeria | 1 | [1] |
| CEC2871 | 12 | blood | invasive | South Korea | 18 | [8] |

*Clades were determined by fingerprint analysis [1, 9–11].

References

[1] F. C. Odds, M.-E. Bougnoux, D. J. Shaw, J. M. Bain, A. D. Davidson, D. Diogo, M. D. Jacobsen, M. Lecomte, S.-Y. Li, A. Tavanti, M. C. J. Maiden, N. A. R. Gow, and C. d’Enfert, “Molecular phylogenetics of Candida albicans.,” *Eukaryot. Cell*, vol. 6, no. 6, pp. 1041–52, Jun. 2007.

[2] W. Wu, S. R. Lockhart, C. Pujol, T. Srikantha, and D. R. Soll, “Heterozygosity of genes on the sex chromosome regulates Candida albicans virulence.,” *Mol. Microbiol.*, vol. 64, no. 6, pp. 1587–604, Jun. 2007.

[3] M. P. Hirakawa, D. A. Martinez, S. Sakthikumar, M. Z. Anderson, A. Berlin, S. Gujja, Q. Zeng, E. Zisson, J. M. Wang, J. M. Greenberg, J. Berman, R. J. Bennett, and C. A. Cuomo, “Genetic and phenotypic intra-species variation in Candida albicans.,” *Genome Res.*, vol. 25, no. 3, pp. 413–25, Mar. 2015.

[4] A. M. Gillum, E. Y. Tsay, and D. R. Kirsch, “Isolation of the Candida albicans gene for orotidine-5’-phosphate decarboxylase by complementation of S. cerevisiae ura3 and E. coli pyrF mutations.,” *Mol. Gen. Genet.*, vol. 198, no. 2, pp. 179–82, 1984.

[5] Y. F. Chan, M. E. Marks, F. C. Jones, G. Villarreal, M. D. Shapiro, S. D. Brady, A. M. Southwick, D. M. Absher, J. Grimwood, J. Schmutz, R. M. Myers, D. Petrov, B. Jónsson, D. Schluter, M. A. Bell, and D. M. Kingsley, “Adaptive evolution of pelvic reduction in sticklebacks by recurrent deletion of a Pitx1 enhancer.,” *Science*, vol. 327, no. 5963, pp. 302–5, Jan. 2010.

[6] M.-E. Bougnoux and C. d’Enfert

[7] C. Angebault, F. Djossou, S. Abélanet, E. Permal, M. Ben Soltana, L. Diancourt, C. Bouchier, P.-L. Woerther, F. Catzeflis, A. Andremont, C. d’Enfert, and M.-E. Bougnoux, “Candida albicans is not always the preferential yeast colonizing humans: a study in Wayampi Amerindians.,” *J. Infect. Dis.*, vol. 208, no. 10, pp. 1705–16, Nov. 2013.

[8] J. H. Shin, M.-E. Bougnoux, C. d’Enfert, S. H. Kim, C.-J. Moon, M. Y. Joo, K. Lee, M.-N. Kim, H. S. Lee, M. G. Shin, S. P. Suh, and D. W. Ryang, “Genetic diversity among Korean Candida albicans bloodstream isolates: assessment by multilocus sequence typing and restriction endonuclease analysis of genomic DNA by use of BssHII.,” *J. Clin. Microbiol.*, vol. 49, no. 7, pp. 2572–7, Jul. 2011.

[9] E. Blignaut, C. Pujol, S. Lockhart, S. Joly, and D. R. Soll, “Ca3 Fingerprinting of Candida albicans Isolates from Human Immunodeficiency Virus-Positive and Healthy Individuals Reveals a New Clade in South Africa,” *J. Clin. Microbiol.*, vol. 40, no. 3, pp. 826–836, Mar. 2002.

[10] C. Pujol, M. Pfaller, and D. R. Soll, “Ca3 Fingerprinting of Candida albicans Bloodstream Isolates from the United States, Canada, South America, and Europe Reveals a European Clade,” *J. Clin. Microbiol.*, vol. 40, no. 8, pp. 2729–2740, Aug. 2002.

[11] S. Lockhart, B. Reed, C. Pierson, and D. Soll, “Most frequent scenario for recurrent Candida vaginitis is strain maintenance with ‘substrain shuffling’: demonstration by sequential DNA fingerprinting with probes Ca3, C1, and CARE2,” *J. Clin. Microbiol.*, vol. 34, no. 4, pp. 767–777, Apr. 1996.
