# Supplementary material for: Transcriptional rewiring over evolutionary timescales changes quantitative and qualitative properties of gene expression
Source: eLife. 2016 Sep 10;5:e18981. doi: 10.7554/eLife.18981 (PMC5067116; doi:10.7554/eLife.18981)
Supplement: Supplementary file 2. — DOI: http://dx.doi.org/10.7554/eLife.18981.024 [file elife-18981-supp2.docx]

**Supplementary File 2: Genes induced by galactose in *S. cerevisiae***

| **Systematic Name** | **Common Name** | **Gal/YEP ratio_1** | **Gal/YEP ratio_2** | **p-value** | **Carbohydrate metabolism annotation** | ***C. albicans* ortholog** |
| --- | --- | --- | --- | --- | --- | --- |
| YAL040C | CLN3 | 2.6 | 4.1 | 3.71E-13 |  | orf19.1960 |
| YBR018C | GAL7 | 626.4 | 1796.1 | 9.89E-83 | yes | orf19.3675 |
| YBR019C | GAL10 | 276.7 | 601.7 | 2.96E-153 | yes | orf19.3672 |
| YBR020W | GAL1 | 354.8 | 797.5 | 2.35E-127 | yes | orf19.3670 |
| YBR238C | YBR238C | 2.3 | 4.1 | 8.2E-15 |  | orf19.7459 |
| YDL048C | STP4 | 5.1 | 12.3 | 2.42E-11 |  |  |
| YDL079C | MRK1 | 4.6 | 12.3 | 2.29E-11 |  |  |
| YDR009W | GAL3 | 19.2 | 15.6 | 5.19E-95 | yes |  |
| YDR010C | YDR010c | 15.9 | 13.5 | 2.25E-31 |  |  |
| YDR277C | MTH1 | 3.6 | 5.2 | 2.91E-42 |  | orf19.6173 |
| YEL057C | YEL057C | 17.5 | 29.4 | 3.94E-33 |  |  |
| YFL002W-B | YFL002W-B | Inf | 482480 | 0.00000541 |  |  |
| YGL034C | YGL034C | 4.7 | 5.9 | 0.0000905 |  |  |
| YGL134W | PCL10 | 2 | 3 | 1.84E-09 | yes |  |
| YGR161W-A | YGR161W-A | Inf | 482480 | 0.00000541 |  |  |
| YGR177C | ATF2 | 2.1 | 3.2 | 7.27E-15 |  | orf19.276 |
| YHR174W | ENO2 | 2.4 | 2.9 | 1.92E-12 | yes |  |
| YIL053W | RHR2 | 2.6 | 4 | 0.00000186 | yes | orf19.5437 |
| YJL157C | FAR1 | 5.9 | 5 | 3.75E-42 |  | orf19.7105 |
| YJL212C | OPT1 | 2.1 | 2.6 | 1.39E-16 |  | orf19.2602 |
| YLL024C | SSA2 | 2.6 | 2.3 | 1.64E-13 |  | orf19.1065 |
| YLR081W | GAL2 | 358.5 | 886.7 | 5.81E-118 | yes |  |
| YML051W | GAL80 | 8.6 | 12.4 | 5.94E-87 | yes |  |
| YNL065W | AQR1 | 2.4 | 2.4 | 1.07E-13 |  |  |
| YNL074C | MLF3 | 3.3 | 4.3 | 5.94E-41 |  |  |
| YOL014W | YOL014W | 4.1 | 4.2 | 6.01E-09 |  |  |
| YOL136C | PFK27 | 2.2 | 4.5 | 0.000000421 | yes | orf19.2308 |
| YOR120W | GCY1 | 4.5 | 5.2 | 1.78E-11 | yes | orf19.6757 |
| YOR121C | YOR121C | 4 | 5.1 | 9.25E-52 |  |  |
| YOR344C | TYE7 | 2.3 | 2.7 | 1.64E-12 | yes | orf19.4941 |
| YPL066W | RGL1 | 8.2 | 9.3 | 7.11E-94 |  | orf19.210 |
| YPL067C | YPL067C | 4.7 | 6.6 | 1.53E-53 |  | orf19.1066 |
| YPL068C | YPL068C | 2.1 | 2.3 | 0.00069202 |  |  |
| YPR194C | OPT2 | 9 | 5.7 | 1.23E-19 |  | orf19.3749 |

* annotated as dubious orfs
