# Supplementary material for: Transcriptional rewiring over evolutionary timescales changes quantitative and qualitative properties of gene expression
Source: eLife. 2016 Sep 10;5:e18981. doi: 10.7554/eLife.18981 (PMC5067116; doi:10.7554/eLife.18981)
Supplement: Supplementary file 3. — DOI: http://dx.doi.org/10.7554/eLife.18981.025 [file elife-18981-supp3.docx]

**Supplementary File 3: Genes induced by galactose in *C. albicans***

| **Systematic Name** | **Common Name** | **Gal /YEP Ratio Expt 1** | **Gal /YEP ratio Expt 2** | **p-value** | **Biofilm annotation** | **Filamentation annotation** | **Pathogenesis annotation** | **Carbohydrate metabolism annotation** | ***S. cerevisiae* ortholog** |
| --- | --- | --- | --- | --- | --- | --- | --- | --- | --- |
| orf19.1415 | FRE10 | 3.4 | 3.2 | 5.62E-09 |  |  |  |  |  |
| orf19.1616 | FGR23 | 5 | 3 | 8.83E-09 |  | yes |  |  |  |
| orf19.1691 | orf19.1681 | 4.5 | 4.8 | 3.15E-107 |  |  |  |  |  |
| orf19.1822 | UME6 | 6.1 | 2.7 | 0.0000289 |  | yes | yes |  |  |
| orf19.2023 | HGT7 | 7.6 | 7.7 | 1.54E-102 |  |  |  |  |  |
| orf19.2308* | orf19.2308 | 4.1 | 2.5 | 1.09E-13 |  |  |  | yes | YOL136C |
| orf19.2376 | orf19.2376 | 3 | 2.2 | 0.00000222 |  |  |  |  |  |
| orf19.2863.1 | ERV1 | 2.5 | 2 | 4.28E-15 |  |  |  |  | YGR029W |
| orf19.3433 | OYE23 | 3.7 | 3.3 | 3.61E-10 |  |  |  |  |  |
| orf19.3670* | GAL1 | 13 | 13.3 | 2.22E-223 |  |  |  | yes | YBR020w |
| orf19.3672* | GAL10 | 9.1 | 10.6 | 2.08E-158 |  | yes |  | yes | YBR019C |
| orf19.3675* | GAL7 | 5.3 | 7 | 6.48E-22 |  |  |  | yes | YBR018C |
| orf19.4215 | FET34 | 3.6 | 4.2 | 0.000000286 |  | yes | yes |  | YMR058W |
| orf19.4318 | MIG1 | 3.7 | 2.6 | 4.3E-25 |  | yes |  |  | YGL035C |
| orf19.4321 | orf19.4321 | Inf | 23.9 | 0.00000251 |  |  |  |  |  |
| orf19.4384 | HXT5 | 6.8 | 3.4 | 7.09E-10 |  |  |  |  |  |
| orf19.4384.1 | orf19.4384.1 | 6.7 | 3.8 | 5.4E-14 |  |  |  |  |  |
| orf19.4899 | GCA1 | 11 | 9.8 | 1.15E-26 | yes |  |  | yes |  |
| orf19.508 | QDR1 | 54.9 | 52.2 | 1.28E-160 | yes |  |  |  | YIL120W |
| orf19.5136 | orf19.5136 | 2.4 | 2.2 | 7.77E-21 |  |  |  |  | YGR017W |
| orf19.5288.1 | orf19.5288.1 | 2.2 | 2 | 4.3E-13 |  |  |  |  |  |
| orf19.5337 | UBC15 | 2.1 | 2.3 | 8.87E-11 |  |  |  |  |  |
| orf19.5392 | NGT1 | 2.9 | 2.1 | 1.53E-12 |  |  |  |  |  |
| orf19.542 | HXK2 | 3.3 | 3.8 | 1.23E-13 |  |  |  | yes | YGL253W |
| orf19.5437* | RHR2 | 2.8 | 2.5 | 9.59E-14 | yes |  | yes | yes | YIL053W |
| orf19.5524 | orf19.5524 | 2.8 | 2.9 | 0.0000164 |  |  |  |  |  |
| orf19.5525 | orf19.5525 | 4.7 | 3.8 | 9.47E-70 |  |  |  |  | YMR315W |
| orf19.5713 | YMX6 | 11.9 | 5.3 | 3.87E-08 |  |  |  |  |  |
| orf19.5902 | RAS2 | 9.8 | 10.9 | 3.11E-15 |  | yes |  |  |  |
| orf19.7094 | HGT12 | 9.5 | 8.6 | 0.000000185 |  | yes |  |  |  |
| orf19.7219 | FTR1 | 2.5 | 2.9 | 1.49E-08 |  |  | yes |  | YER145C |
| orf19.7585 | INO1 | 7.3 | 17.1 | 3.18E-08 |  |  |  | yes | YJL153C |
| orf19.999 | GCA2 | 5.6 | 3.1 | 7.35E-13 | yes |  |  | yes |  |

*orthologs induced by galactose in *S. cerevisiae*
