# Supplementary material for: Transcriptional rewiring over evolutionary timescales changes quantitative and qualitative properties of gene expression
Source: eLife. 2016 Sep 10;5:e18981. doi: 10.7554/eLife.18981 (PMC5067116; doi:10.7554/eLife.18981)
Supplement: Supplementary file 4. — DOI: http://dx.doi.org/10.7554/eLife.18981.026 [file elife-18981-supp4.docx]

**Supplementary File 4: List of *C. albicans* strains used in this study**

| **Strain Number** | **Genotype** | **Used in Figures (Annotation)** | **Reference** |
| --- | --- | --- | --- |
| OH13 | *arg4∆/arg4∆ leu2∆/leu2∆::CmLEU2 his1∆/his1∆::CdHIS1 URA3/ura3∆::imm^434^ IRO1/iro1∆::imm^434^* | 1b 1S1a 3a 1S1b 5 5S1 5S2  (Parent strain) | [1] |
| JCP161 | *arg4∆/arg4∆ leu2∆/leu2∆ his1∆/his1∆ URA3/ura3∆::imm^434^ IRO1/iro1∆::imm^434^ orf19.3670∆::CdHis1/orf19.3670∆::CmLeu2* | 1b 1S1a 3a 1S1b (∆/∆*gal1*) | [2] |
| CD158 | *arg4∆/arg4∆ leu2∆/leu2∆ his1∆/his1∆ URA3/ura3∆::imm^434^ IRO1/iro1∆::imm^434^ orf19.3675∆::CdHis1/orf19.3675∆::CmLeu2* | 1b 1S1a  (∆/∆*gal7*) | this work |
| JCP148 | *arg4∆/arg4∆ leu2∆/leu2∆ his1∆/his1∆ URA3/ura3∆::imm^434^ IRO1/iro1∆::imm^434^ orf19.3672∆::CdHis1/orf19.3672∆::CmLeu2* | 1b 1S1a (∆/∆*gal10*) | [2] |
| CD008 | *arg4∆/arg4∆ leu2∆/leu2∆::CmLEU2 his1∆/his1∆::CdHIS1 URA3/ura3∆::imm^434^ IRO1/iro1∆::imm^434^ orf19.3670::GFP-SAT1/orf19.3670* | 1c 3c 6a  (Parent strain) | this work |
| CD017 | *arg4∆/arg4∆ leu2∆/leu2∆ his1∆/his1∆ URA3/ura3∆::imm^434^ IRO1/iro1∆::imm^434^ orf19.3670∆::CdHis1/orf19.3670::GFP-SAT1* | 1c  (∆/∆*gal1*) | this work |
| CD016 | *arg4∆/arg4∆ leu2∆/leu2∆ his1∆/his1∆ URA3/ura3∆::imm^434^ IRO1/iro1∆::imm^434^ orf19.6899∆::CdHis1/orf19.6899∆::CmLeu2 orf19.3670::GFP-SAT1/orf19.3670* | 1c  (∆/∆*gal80*) | this work |
| SN425 | *arg4:CdARG4/arg4∆ leu2∆/leu2∆::CmLEU2 his1∆/his1∆::CdHIS1 URA3/ura3∆::imm^434^ IRO1/iro1∆::imm^434^* | 2a 2Sa  (Parent strain) | [3] |
| CD151 | *arg4∆:CdARG4/arg4∆ leu2∆/leu2∆ his1∆/his1∆ URA3/ura3∆::imm^434^ IRO1/iro1∆::imm^434^ orf19.3670∆::CdHis1/orf19.3670∆::CmLeu2* | 2b 2Sb  (∆/∆*gal1*) | this work |
| CD157 | *arg4∆:CdARG4/arg4∆ leu2∆/leu2∆ his1∆/his1∆ URA3/ura3∆::imm^434^ IRO1/iro1∆::imm^434^ orf19.3675∆::CdHis1/orf19.3675∆::CmLeu2* | 2c 2Sc  (∆/∆*gal7*) | this work |
| CD153 | *arg4∆:CdARG4/arg4∆ leu2∆/leu2∆ his1∆/his1∆ URA3/ura3∆::imm^434^ IRO1/iro1∆::imm^434^ orf19.3672∆::CdHis1/orf19.3672∆::CmLeu2* | 2d 2Sd (∆/∆*gal10*) | this work |
| TF045 | *arg4∆/arg4∆ leu2∆/leu2∆ his1∆/his1∆ URA3/ura3∆::imm^434^ IRO1/iro1∆::imm^434^ orf19.5338∆::CdHis1/orf19.5338∆::CmLeu2* | 3a 1Sb  (∆/∆*gal4*) | [1] |
| CD035 | *arg4∆/arg4∆ leu2∆/leu2∆::CmLEU2 his1∆/his1∆::CdHIS1 URA3/ura3∆::imm^434^ IRO1/iro1∆::imm^434^ orf19.5338∆-FRT/orf19.5338∆-FRT* | 3a 1Sb  (∆/∆*cph1*) | this work |
| TF034 | *arg4∆/arg4∆ leu2∆/leu2∆ his1∆/his1∆ URA3/ura3∆::imm^434^ IRO1/iro1∆::imm^434^ orf19.4772∆::CdHis1/orf19.4772∆::CmLeu2* | 3a 1Sb  (∆/∆*rtg1*) | [1] |
| JCP194 | *arg4∆/arg4∆ leu2∆/leu2∆ his1∆/his1∆ URA3/ura3∆::imm^434^ IRO1/iro1∆::imm^434^ orf19.4772∆::CdHis1/orf19.4772∆::CmLeu2 rps1∆::orf19.4772-SAT1* | 3a 1Sb  (∆/∆*rtg1* + *RTG1*) | [2] |
| TF142 | *arg4∆/arg4∆ leu2∆/leu2∆ his1∆/his1∆ URA3/ura3∆::imm^434^ IRO1/iro1∆::imm^434^ orf19.2315∆::CdHis1/orf19.2315∆::CmLeu2* | 3a 1Sb  (∆/∆*rtg3*) | [1] |
| JCP191 | *arg4∆/arg4∆ leu2∆/leu2∆ his1∆/his1∆ URA3/ura3∆::imm^434^ IRO1/iro1∆::imm^434^ orf19.2315∆::CdHis1/orf19.2315∆::CmLeu2 rps1∆::orf19.2315-SAT1* | 3a 1Sb  (∆/∆*rtg3* + *RTG3*) | [2] |
| TFKO | *arg4∆/arg4∆ leu2∆/leu2∆ his1∆/his1∆ URA3/ura3∆::imm^434^ IRO1/iro1∆::imm^434^ TF∆::CdHis1/TF∆::CmLeu2* | 3b  (all listed in Figure 3- Source Data) | [1] [4] |
| GTC43 (TF184) | *ura3/ura3∆::imm^434^ gcn4::hisG‐URA3‐hisG/gcn4::hisG* | 3b  (∆/∆*gcn4*) | [5] |
| CD188 | *arg4∆/arg4∆ leu2∆/leu2∆::CmLEU2 his1∆/his1∆::CdHIS1 URA3/ura3∆::imm^434^ IRO1/iro1∆::imm^434^ orf19.4772∆::CdHis1/orf19.4772∆::CmLeu2 orf19.3670::GFP-SAT1/orf19.3670* | 3c  (∆/∆*rtg1*) | this work |
| CD191 | *arg4∆/arg4∆ leu2∆/leu2∆::CmLEU2 his1∆/his1∆::CdHIS1 URA3/ura3∆::imm^434^ IRO1/iro1∆::imm^434^ orf19.4772∆::CdHis1/orf19.4772∆::CmLeu2 orf19.3670::GFP-SAT1/orf19.3670* | 3c  (∆/∆*rtg3*) | this work |
| CD247 | *arg4∆/arg4∆ leu2∆/leu2∆::CmLEU2 his1∆/his1∆::CdHIS1 URA3/ura3∆::imm^434^ IRO1/iro1∆::imm^434^ orf19.4772∆::CdHis1/orf19.4772∆::CmLeu2 orf19.2315∆-FRT/orf19.2315∆-FRT orf19.3670::GFP-SAT1/orf19.3670* | 3c  (∆/∆*rtg1* ∆/∆*rtg3*) | this work |
| CD421 | *arg4∆/arg4∆ leu2∆/leu2∆::CmLEU2 his1∆/his1∆::CdHIS1 URA3/ura3∆::imm^434^ IRO1/iro1∆::imm^434^ rps1∆::Cyc1p∆UAS-2xbindingsites-GFP-SAT1* | 3d 6b  (Parent) | this work |
| CD424 | *arg4∆/arg4∆ leu2∆/leu2∆::CmLEU2 his1∆/his1∆::CdHIS1 URA3/ura3∆::imm^434^ IRO1/iro1∆::imm^434^ orf19.4772∆::CdHis1/orf19.4772∆::CmLeu2 orf19.2315∆-FRT/orf19.2315∆-FRT rps1∆::Cyc1p∆UAS-2xbindingsites-GFP-SAT1* | 3d 6b  (∆/∆rtg1 ∆/∆rtg3) | this work |
| CD171 | *arg4∆/arg4∆ C.m.LEU2/leu2∆ C.d.HIS1/his1∆ URA3/ura3∆::imm^434^ IRO1/iro1∆::imm^434^ orf19.3670::GFP-SAT1/orf19.3670 RPL26B-mCherry/RPL26B* | 4 4S1 4S2  (*C. albicans* SC5314*)* | this work |
| Clinical Isolates | Clinical isolates *orf19.3670::GFP-SAT1/orf19.3670* | 4S2  (Isolates listed in Supplementary File 1) | this work |

References

[1] O. R. Homann, J. Dea, S. M. Noble, and A. D. Johnson, “A phenotypic profile of the Candida albicans regulatory network.,” *PLoS Genet.*, vol. 5, no. 12, p. e1000783, Dec. 2009.

[2] J. C. Perez, C. A. Kumamoto, and A. D. Johnson, “Candida albicans Commensalism and Pathogenicity Are Intertwined Traits Directed by a Tightly Knit Transcriptional Regulatory Circuit,” *PLoS Biol*, vol. 11, no. 3, p. e1001510, 2013.

[3] S. M. Noble, S. French, L. A. Kohn, V. Chen, and A. D. Johnson, “Systematic screens of a Candida albicans homozygous deletion library decouple morphogenetic switching and pathogenicity.,” *Nat. Genet.*, vol. 42, no. 7, pp. 590–8, Jul. 2010.

[4] E. P. Fox, E. S. Cowley, C. J. Nobile, N. Hartooni, D. K. Newman, and A. D. Johnson, “Anaerobic bacteria grow within Candida albicans biofilms and induce biofilm formation in suspension cultures.,” *Curr. Biol.*, vol. 24, no. 20, pp. 2411–6, Oct. 2014.

[5] G. Tripathi, C. Wiltshire, S. Macaskill, H. Tournu, S. Budge, A. J. P. Brown, G. Albrecht, H. Mosch, B. Hoffman, U. Reusser, G. Braus, K. Arndt, G. Fink, G. Bertram, R. Swoboda, N. Gow, G. Gooday, A. Brown, D. Bockmuhl, J. Ernst, D. Bohmann, T. Bos, A. Admon, T. Nishimura, P. Vogt, R. Tjian, B. Braun, A. Johnson, B. Braun, D. Kadosh, A. Johnson, A. Brown, N. Gow, A. Brown, D. Brown, A. Giusani, X. Chen, C. Kumamoto, R. Care, J. Trevethick, K. Binley, P. Sudbery, B. Cormack, G. Bertram, M. Egerton, N. Gow, S. Falkow, A. Brown, D. Davis, R. Wilson, A. Mitchell, C. Drysdale, E. Dueñas, B. Jackson, U. Reusser, G. Braus, A. Hinnebusch, D. Ebbole, J. Paluh, M. Plamann, M. Sachs, C. Yanofsky, A. El Barkani, O. Kurzai, W. Fonzi, A. Ramon, A. Porta, M. Frosch, F. Muhlschlegel, D. Engelberg, C. Klein, H. Martinetto, K. Struhl, M. Karin, J. Ernst, Q. Feng, E. Summers, B. Guo, G. Fink, W. Fonzi, M. Irwin, J. Gancedo, R. Gietz, R. Woods, A. Gillum, E. Tsay, D. Kirsch, A. Hinnebusch, D. Kadosh, A. Johnson, C. Keleher, M. Redd, J. Schultz, M. Carlson, A. Johnson, S. Kron, S. Lane, S. Zhou, T. Pan, Q. Dai, H. Liu, S. Lane, C. Birse, S. Zhou, R. Matson, H. Liu, E. Leberer, D. Harcus, D. Dignard, L. Johnson, S. Ushinsky, D. Thomas, K. Schroeppel, K. Lengeler, R. Davidson, C. D’Souza, T. Harashima, W. Shen, P. Wang, X. Pan, M. Waugh, J. Heitman, H. Liu, J. Kohler, G. Fink, H. Lo, J. Kohler, B. DiDomenico, D. Loebenberg, A. Cacciapuoti, G. Fink, Z. Luo, M. Freitag, M. Sachs, M. Marton, A. Murad, P. Lee, I. Broadbent, C. Barelle, A. Brown, A. Murad, K. Natarajan, M. Meyer, B. Jackson, D. Slade, C. Roberts, A. Hinnebusch, M. Marton, F. Odds, B. Osborne, L. Guarente, J. Paluh, M. Orbach, T. Legerton, C. Yanofsky, S. Pereira, G. Livi, A. Ramon, A. Porta, W. Fonzi, M. Schaller, W. Schafer, H. Korting, B. Hube, D. Smith, M. Cooper, M. DeTiani, C. Losberger, M. Payton, T. Srikantha, A. Klapach, W. Lorenz, L. Tsai, L. Laughlin, J. Gorman, D. Soll, V. Stoldt, A. Sonneborn, C. Leuker, J. Ernst, P. Sudbery, R. Swoboda, G. Bertram, S. Delbruck, J. Ernst, N. Gow, G. Gooday, A. Brown, B. Thomas, R. Rothstein, K. Tzung, J. van Helden, B. André, J. Collado‐Vides, C. Wanke, S. Eckert, G. Albrecht, W. van Hartingsveldt, P. Punt, C. van den Hondel, G. Braus, M. Whiteway, C. Wiltshire, S. Black, and A. Brown, “Gcn4 co-ordinates morphogenetic and metabolic responses to amino acid starvation in Candida albicans.,” *EMBO J.*, vol. 21, no. 20, pp. 5448–56, Oct. 2002.
